# Early Use of Corticosteroids following CAR T-Cell Therapy Correlates with Reduced Risk of High-Grade CRS without Negative Impact on Neurotoxicity or Treatment Outcome

**DOI:** 10.3390/biom13020382

**Published:** 2023-02-17

**Authors:** Tim Lakomy, Dilara Akhoundova, Henning Nilius, Marie-Noëlle Kronig, Urban Novak, Michael Daskalakis, Ulrike Bacher, Thomas Pabst

**Affiliations:** 1 Department of Medical Oncology, Inselspital, Bern University Hospital, University of Bern, 3012 Bern, Switzerland; 2 University Institute of Clinical Chemistry, Inselspital, Bern University Hospital, University of Bern, 3010 Bern, Switzerland; 3 Department of Hematology and Central Hematology Laboratory, Inselspital, Bern University Hospital, University of Bern, 3010 Bern, Switzerland

**Keywords:** CAR T-cell therapy, adverse events, CRS, neurotoxicity, ICANS, tocilizumab, corticosteroids

## Abstract

Background: Chimeric antigen receptor T-cell therapy (CAR T-cell therapy) is associated with potentially life-threatening toxicities, most commonly cytokine release syndrome (CRS) and immune-effector-cell-associated neurotoxicity syndrome (ICANS). These frequent adverse events are managed with the IL-6 receptor antagonist tocilizumab and/or corticosteroids. The prophylactic and early use of corticosteroids for CRS and ICANS have previously been reported, but eventual negative impacts on CAR T-cell efficacy are feared. Methods: Retrospective comparative analysis of two patient cohorts with hematological malignancies treated with CAR T-cell therapy: 43 patients received early administration of 10 mg dexamethasone preceding each dose of tocilizumab (“early corticosteroid/ tocilizumab”, EcsTcz cohort) vs. 40 patients who received tocilizumab alone (“tocilizumab alone”, Tcz cohort) for treatment of low-grade CRS. Results: Despite overall higher CRS incidence (91% vs. 70%; *p* = 0.0249), no high-grade CRS was observed (0% vs. 10%; *p* = 0.0497) among patients receiving early corticosteroids in combination with tocilizumab. In terms of neurotoxicity, no worsening regarding incidence of ICANS (30% vs. 33%; *p* = 0.8177) or high-grade ICANS (20% vs. 14%; *p* = 0.5624) was observed in the EcsTcz cohort. Moreover, overall response rates (80% vs. 77%; *p* = 0.7936), complete response rates (50% vs. 44%; *p* = 0.6628), progression-free survival (*p* = 0.6345) and overall survival (*p* = 0.1215) were comparable for both cohorts. Conclusions: Our study suggests that the early use of corticosteroids in combination with the standard tocilizumab schedule for low-grade CRS following CAR T-cell therapy may significantly reduce the risk of high-grade CRS without negative impact on neurotoxicity or treatment outcome.

## 1. Introduction

Chimeric antigen receptor T-cell therapy (CAR T-cell therapy) is a rapidly emerging cellular immunotherapy approach which has revolutionized the management of relapsed/refractory (r/r) hematological malignancies [1]. Currently approved CAR T-cell products target CD19 and B-cell maturation antigen (BCMA), allowing successful treatment and unprecedented responses in r/r diffuse large B-cell lymphoma (DLBCL), r/r B-cell acute lymphoblastic leukemia (B-ALL), r/r mantle cell lymphoma (MCL) and, more recently, r/r multiple myeloma [1,2]. Overall response rates (ORRs) and complete response rates (CRRs) range from 52% to 93% and 40% to 67%, respectively [3,4,5,6,7,8,9]. For patients with DLBCL or MCL and persistent disease after CAR T-cell therapy, the bispecific antibody glofitamab targeting CD20 and CD3 has been shown to be well tolerated, leading to significant tumor responses, and may enhance residual CAR T-cell activity [10,11].

However, CAR T-cell therapy is associated with unique toxicities, such as cytokine release syndrome (CRS) and immune-effector-cell-associated neurotoxicity syndrome (ICANS) [12]. The incidence of CRS ranges from 42% to 94%, and for high-grade (grades 3 and 4) CRS, from 6% to 25%. ICANS has been reported in 21% to 64% of cases, with a frequency of 3% to 31% for high-grade events [3,4,5,6,7,8,9]. Occurrence of higher-grade CRS has been associated with high disease burden and younger age, second-generation CAR T-cell products, and higher dose of administered CAR T-cells [13]. Moreover, cyclophosphamide- and fludarabine-based lymphodepleting regimens have been correlated with higher CRS incidence [13]. Relevantly, high-grade ICANS has been associated with early and/or high-grade CRS, as well as pre-existing neurologic comorbidities [14]. Current management of CRS is based on early administration of the monoclonal antibody tocilizumab, targeting the IL-6 receptor, the use of which is indicated in grade 2 or higher CRS. In more severe cases, refractory grade 2 and grade 3 or higher events, concomitant use of corticosteroids is recommended [15]. Moreover, siltuximab, an anti-IL-6 monoclonal antibody, may be used in case of refractory CRS. On the contrary, ICANS therapy without concurrent CRS, is based on corticosteroids, since tocilizumab passes the blood–brain barrier poorly [15,16,17,18,19,20,21,22]. For successful management of both adverse events, active screening and early detection are essential [15].

A new approach to CRS and ICANS management is the prophylactic and/or early use of corticosteroids following CAR T-cell infusion. This approach has been shown to lower CRS incidence, lower high-grade CRS rates and shorten the duration of CRS symptoms. In terms of neurotoxicity, no increased risk of ICANS and, in some cases, lower incidence, severity and duration of ICANS have been reported [7,17,18,23]. With the prophylactic and/or early use of corticosteroids, possible negative impacts on the efficacy of CAR T-cell therapy are feared due to possible suppression of CAR T-cell activity. However, no impact on outcome has been observed yet with any use of corticosteroids following CAR T-cell therapy [7,17,18,23,24,25]. In this study, we aimed to assess the consequences of the early use of corticosteroids in combination with standard tocilizumab administration for CAR T-cell-therapy-related CRS on the incidence of high-grade CRS, the incidence and severity of ICANS, and CAR T-cell treatment efficacy.

## 2. Materials and Methods

### 2.1. Patient Cohorts and Study Design

We performed a retrospective analysis of all consecutive patients with DLBCL and B-ALL who were treated with CAR T-cell therapy at the University Hospital of Bern, Switzerland, between January 2019 and August 2022. We included all patients with a diagnosis of DLBCL and B-ALL treated with tisagenlecleucel (Kymriah^®^) and axicabtagene-ciloleucel (Yescarta^®^). We excluded patients who were planned to receive CAR T-cell therapy but progressed or presented clinical deterioration before CAR T-cell infusion. Furthermore, patients diagnosed with MCL treated with brexucabtagene-autoleucel (Tecartus^®^) were excluded as well due to expected higher rates of CRS and ICANS [6,7].

**Figure 1:**
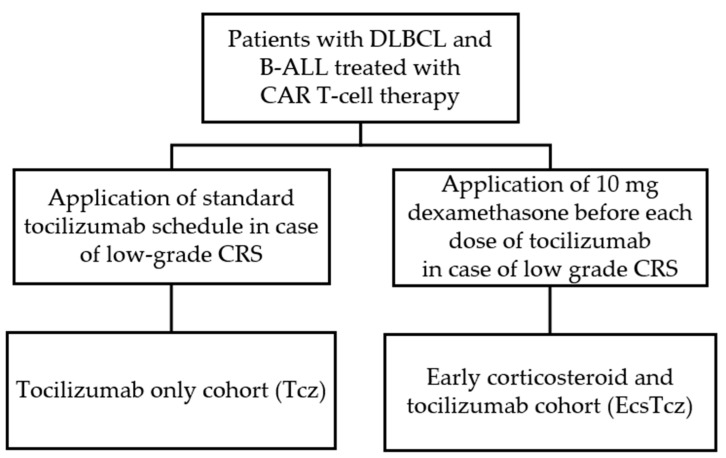
Schematic illustration of study design and patient cohorts. Abbreviations: B-ALL: B-cell acute lymphoblastic leukemia; CAR-T cell therapy: chimeric antigen receptor T-cell therapy; CRS: cytokine release syndrome; DLBCL: diffuse large B-cell lymphoma.

The patients included in the analysis were retrospectively divided into two cohorts for comparative analysis. Patients treated before September 2020 who received treatment for low-grade CRS according to the local standard tocilizumab schedule but without early steroid application were assigned to the “tocilizumab only” (Tcz) cohort. Due to a change in institutional practice guidelines, patients treated after September 2020 received intravenous applications of 10 mg dexamethasone (early corticosteroids) before each dose of tocilizumab in case of low-grade CRS and were assigned to the “early corticosteroid and tocilizumab” (EcsTcz) cohort (Figure 1).

### 2.2. Patient Stratification, Adverse Event Grading and Response Assessment

Patients with DLBCL were stratified by disease stage following the Ann Arbor staging system. Grading of CRS and ICANS was performed according to the ASTCT consensus grading for cytokine release syndrome and neurologic toxicity associated with immune effector cells [12]. Corticosteroid application was distinguished between early application of corticosteroids before the application of tocilizumab for low-grade CRS (early corticosteroids) and subsequent corticosteroid therapy for treatment of refractory or high-grade CRS and ICANS after tocilizumab application (subsequent corticosteroids). Neurological CARTOX-10 assessment was performed twice daily in all patients, as recommended by the CAR T-cell-therapy-associated toxicity (CARTOX) working group [26]. Rebound CRS and ICANS were defined as recurrent events after previous complete remission. Response status to CAR-T cell therapy was classified as: complete response (CR), partial response (PR), stable disease (SD) and progressive disease (PD). Very good partial response was assigned to the partial response category. Overall response rate (ORR) was defined as the proportion of patients who achieved CR or PR, and complete response rate (CRR) as the proportion of patients with CR. Response was assessed using radiological criteria and was based on computer topographies (CTs) performed one month after CAR T-cell infusion, as well as positron emission tomographies (PET-CTs) performed three, six and twelve months after CAR T-cell infusion. Laboratory values collected only during the hospitalization period for CAR T-cell therapy were included in the analysis. However, for the CAR T-cell-expansion measurements, all values up to the collection deadline were included.

### 2.3. Endpoints and Statistical Analysis

The primary endpoints of this study were incidence and severity of CRS and ICANS following CAR T-cell therapy in the EcsTcz vs. Tcz cohorts. Secondary endpoints were overall response rate (ORR), complete response rate (CRR), progression-free survival (PFS) and overall survival (OS).

Patient baseline characteristics, therapy-related data, laboratory values, adverse events and therapy outcome data were retrospectively collected for both cohorts. As the follow-up was shorter for the EcsTcz cohort, an additional PFS and OS analysis with a 12-month data cut-off (PFS 12 months, OS 12 months) was performed. Furthermore, correlations of multiple predictors with CRS and ICANS incidence, as well as PFS and OS, were calculated.

For categorical data, Fisher’s exact test was used. The unpaired *t*-test was applied for normally distributed metrical data. In case of not normally distributed metrical data, the Mann–Whiney *U* test was used. PFS and OS were analyzed with Kaplan–Meier analysis and the Mantel–Cox test. For PFS calculations, events were defined as disease progression or death, whereas for OS death only was considered for event definition. For the calculation of the associations of predictors with categorical target events, multivariable Cox regression was performed. *p*-values lower than 0.05 were considered significant. Descriptive statistics, Kaplan–Meier curves, the calculation of *p*-values and the creation of figures were conducted with GraphPad Prism 9.0.1 for Windows (GraphPad Software, San Diego, California USA). Multivariable analysis was performed with R version 4.1.2 [27].

## 3. Results

### 3.1. Patient Baseline Characteristics

In total, 83 patients were included in the study. Forty patients, who were treated before September 2020 and received tocilizumab only for CAR T-cell-therapy-induced low-grade CRS, were assigned to the Tcz cohort. Forty-three patients treated after September 2020, who received early corticosteroid combined with tocilizumab applications, were allocated to the EcsTcz cohort. Based on predefined exclusion criteria, eight additional patients were excluded: three patients with MCL treated with brexucabtagene-autoleucel and five patients who died before CAR T-cell reinfusion. A significantly higher proportion of patients in the Tcz cohort received stem-cell transplantation (SCT) (68% vs. 37%; *p* = 0.0083). Most SCTs were autologous stem-cell transplantations (ASCTs) (60% vs. 35%; *p* = 0.0285). Otherwise, both cohorts were comparable regarding baseline clinical characteristics. No significant differences in potential risk factors for the development of CRS and ICANS were observed, such as disease stage, remission status before CAR T-cell therapy, central nervous system involvement (10 vs. 16%; *p* = 0.5226) or LDH elevation before CAR T-cell therapy (23 vs. 12%; *p* = 0.2448). The patient baseline characteristics are summarized in Table 1.

### 3.2. Therapy and Laboratory Values

Seventy-two percent of patients in the EcsTcz cohort received early corticosteroid applications, as they developed relevant CRS (72% vs. 0%; *p* > 0.0001) (Table 2). Patients in the EcsTcz cohort received tocilizumab more frequently (74% vs. 50%; *p* = 0.0253), with a comparable median number of applications (4 vs. 4; *p* = 0.1017) but a lower median number of cumulative doses (2400 mg vs. 3200 mg; *p* = 0.0054) than patients in the Tcz cohort. Following the change in institutional practice beginning in December 2019, neutropenia prophylaxis with granulocyte-colony-stimulating factors (G-CSFs) was regularly applied. Thus, a significantly higher use of G-CSFs was registered for the EcsTcz vs. the Tcz cohort (100% vs. 63%; *p* < 0.0001). Laboratory parameters assessed during and after hospitalization for CAR T-cell therapy did not differ significantly between both cohorts.

### 3.3. CRS

**Figure 2:**
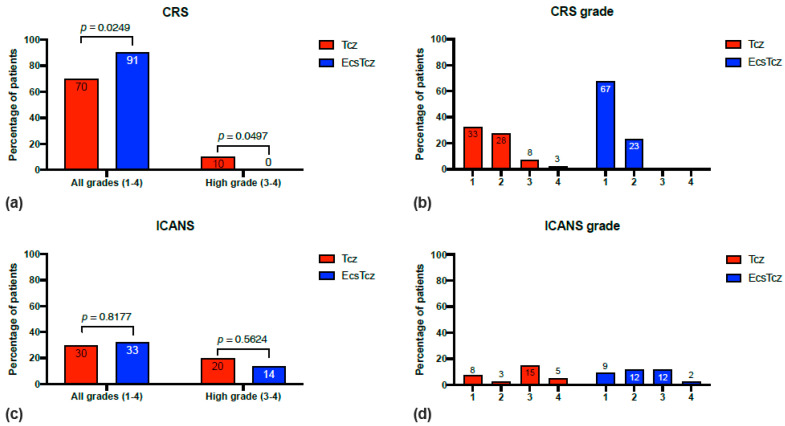
Comparison of adverse events from CAR T-cell therapy in cohorts Tcz vs. EcsTcz. (**a**) Comparison of CRS incidence. (**b**) Visualization of CRS grade distribution. (**c**) Comparison of ICANS incidence. (**d**) Visualization of ICANS grade distribution.

A significantly lower incidence of all CRS grades (grades 1 to 4) in cohort Tcz vs. cohort EcsTcz (70% vs. 91%; *p* = 0.0249) was observed (Table 3; Figure 2). However, despite a higher all-grade CRS frequency, 10% of patients in the Tcz cohort developed high-grade (grades 3 and 4) CRS, while no cases of high-grade CRS were reported in the EcsTcz cohort (10% vs. 0%; *p* = 0.0497). More detailed analysis of the CRS grade distribution showed that the Tcz cohort had a significantly lower rate of grade 1 CRS (33% vs. 67%; *p* = 0.0021). For all other analyzed parameters, both cohorts were comparable, in particular, respecting the rate of intensive care unit transfers (20 vs. 12%; *p* = 0.3709) and duration of hospitalization (22 vs. 21%; *p* = 0.7611). In the multivariable analysis (Table 4), patients who received G-CSFs showed an increased risk for CRS of any degree (OR 10.29; *p* = 0.0107).

### 3.4. ICANS

No worsening in the incidence of all-grade (grades 1 to 4) ICANS (30% vs. 33%; *p* = 0.8177) or high-grade (grades 3 and 4) ICANS (20% vs. 14%; *p* = 0.5624) was observed in patients receiving early corticosteroids and tocilizumab. Accordingly, both cohorts showed a similar frequency of the lowest CARTOX 10 scores (CARTOX 10 scores are included in ICANS grading). In the multivariable analysis (Table 4), higher age was the only significant predictor associated with an increased odds ratio (OR) for ICANS (OR 10.16; *p* = 0.0123). All other predictors showed no significant correlation with CRS or ICANS, including the used CAR T-cell product. The application of early corticosteroids did not increase the OR for ICANS significantly (OR 0.75; *p* = 0.7562).

### 3.5. Outcomes

**Figure 3:**
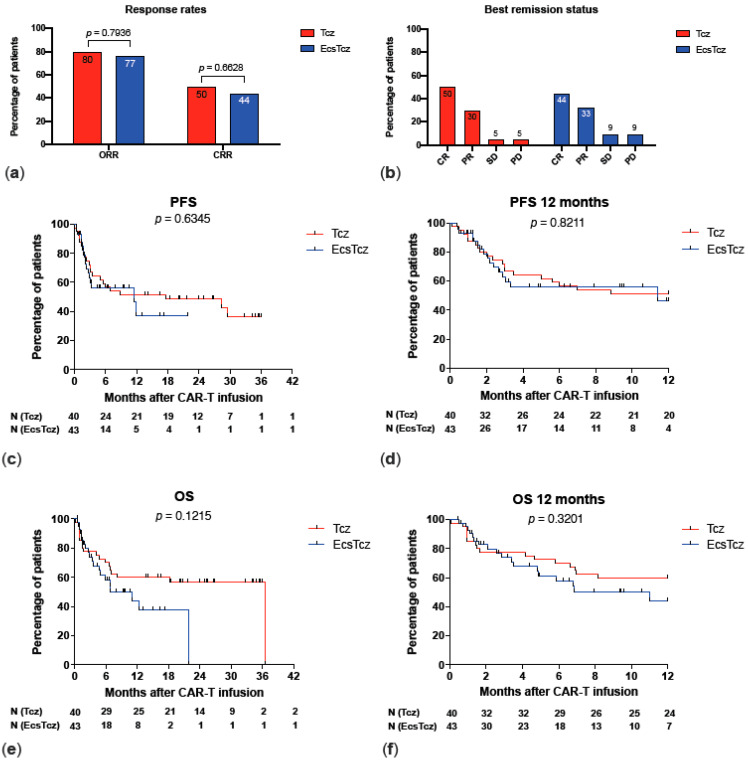
Comparison of outcomes of CAR T-cell therapy in cohorts Tcz vs. EcsTcz. (**a**) Comparison of overall response rates (ORRs) and complete remission rates (CRRs). (**b**) Distribution of best remission status. (**c**) Progression-free survival (PFS). (**d**) Progression-free survival in the first 12 months. (**e**) Overall survival. (**f**) Overall survival in the first 12 months.

In terms of best response and remission status at last follow-up, the cohorts did not show any significant differences. ORRs (80% vs. 77%; *p* = 0.7936) and CRRs (50% vs. 44%; *p* = 0.6628) were comparable for both cohorts, Tcz and EcsTcz (Table 5; Figure 3). Relevantly, no significant differences were observed for PFS (*p* = 0.6345) and OS (*p* = 0.1215), as well as for PFS at 12 months (*p* = 0.8221) and OS at 12 months (*p* = 0.1842). Median follow-up was shorter for the EcsTcz cohort due to chronologically later treatment dates. In the multivariable analysis (Table 6), PFS was significantly shorter for patients of male sex (HR 3.35; *p* = 0.0134) and with G-CSFs (HR 5.09; *p* = 0.0348) and higher serum ferritin peaks (HR 2.91; *p* = 0.0430). On the contrary, patients with higher initial disease stage at diagnosis showed longer PFS (HR 0.62; *p* = 0.0475). Additionally, the use of axicabtagene-ciloleucel (Yescarta^®^) correlated with better OS (HR 0.31; *p* = 0.0122), as compared to tisagenlecleucel (Kymriah^®^). All other predictors showed no significant correlation with PFS or OS. Importantly, the early use of corticosteroids showed no impact on PFS (HR 0.56; *p* = 0.2737) or OS (HR 1.29; *p* = 0.6531).

## 4. Discussion

CRS and ICANS are potentially life-threatening adverse events of CAR T-cell therapy which have negative impacts on patients’ quality of life [28,29]. CRS first-line therapy relies on a combination of tocilizumab and corticosteroids, while isolated ICANS is currently managed mainly with corticosteroids [15,16]. For refractory CRS, siltuximab and anakinra constitute emerging treatment options [29]. While tocilizumab targets the IL-6 receptor, siltuximab is a monoclonal antibody that binds directly to the circulating IL-6 [30]. Anakinra is an IL-1-receptor antagonist that can be used for refractory CRS and ICANS [29,31]. Other potential treatment options, based on small series or case reports, are ruxolitinib [32], a JAK inhibitor; dasatinib, a tyrosine kinase inhibitor [33,34]; and cyclophosphamide [31]. Due to a key role of activated host macrophages in the pathogenesis of CRS, the mechanism of action of corticosteroids relies on inhibition of this excessive immune response [35,36]. Following in vivo interaction of CAR T- and tumor cells, CAR T-cells liberate inflammatory cytokines (e.g., TNF-α and IFN-γ), leading to the hyperactivation of macrophages and the secretion of large amounts of IL-6 and IL-1β [36], which can be effectively abolished by the use of corticosteroids.

Current guidelines recommend the addition of dexamethasone for grade 2 CRS refractory to at least one dose of tocilizumab, as well as for grade 3 or higher CRS [15,37]. Some previous studies have shown that prophylactic and/or early use of corticosteroids might be a promising strategy to prevent the occurrence of high-grade CRS [7,17,18,23]. The use of prophylactic and/or early dexamethasone has been prospectively evaluated in cohort 6 of the ZUMA-1 trial, assessing the safety and efficacy of axicabtagene-ciloleucel in r/r DLBCL [18]. Data from this cohort suggested a positive impact of prophylactic and early use of corticosteroids on the incidence of high-grade CRS, with no impact on the incidence of ICANS or on therapy outcome [18]. However, this approach has not yet been implemented in the current guidelines, and possible negative impacts of corticosteroids on the efficacy of CAR T-cell therapy are still feared [7,17,18,23,25]. Our study aimed to evaluate the effects of the early use of dexamethasone for low-grade CRS in combination with standard tocilizumab on the emergence of higher-grade CRS and all-grade ICANS following CAR T-cell therapy. Moreover, we aimed to determine possible negative impacts on CAR T-treatment efficacy.

In this retrospective observational study, we analyzed a cohort of 83 patients (94% DLBCL, 6% B-ALL), stratified by early use of dexamethasone for low-grade CRS in addition to tocilizumab. Both cohorts were comparable regarding size and patient baseline characteristics. The only significant difference was a higher percentage of patients with a history of high-dose chemotherapy (HDCT) and ASCT in the Tcz vs. the EcsTcz cohort (60% vs. 35%; *p* = 0.0285). Since the Tcz cohort consisted of patients treated before September 2020, a higher proportion of patients received CAR T-cell therapy in later treatment lines, including previous treatment consolidation with HDCT and ASCT. Notably, the proportion of patients with transformed vs. de novo DLBCL showed no significant difference (43% vs. 28%; *p* = 0.1765). This is prognostically relevant, since transformed DLBCL has been associated with better CAR T-cell therapy outcome [38].

Our study showed that no high-grade CRS occurred in the patient cohort receiving prophylactic steroids with each tocilizumab dose (EcsTcz cohort), whereas 10% of patients in the Tcz cohort developed grade 3–4 CRS (*p* = 0.0497). This was despite a higher overall frequency of CRS in the EcsTcz cohort (70% vs. 91%; *p* = 0.0249), with the additional events corresponding mainly to grade 1 CRS (33% vs. 67%; *p* = 0.0021). This finding suggests a significant reduction in progression from low- to high-grade CRS with the early use of corticosteroids, supporting previously published data [17,18,23]. Since early corticosteroids were administered only to patients in which CRS had already occurred, the additional low-grade CRS cases in cohort EcsTcz were attributed to the more frequent use of G-CSFs (63% vs. 100%; *p* < 0.0001), among other possible factors. This assumption was supported by the fact that the use of G-CSFs was a significant predictor for CRS in the multivariable analysis (OR 10.29; *p* = 0.0107). Moreover, the use of G-CSFs following CAR T-cell therapy has been related to increased CRS severity in previous work [39]. However, the available data are insufficient to support this association.

In terms of neurotoxicity, no worsening in the incidence of ICANS (30% vs. 33%; *p* = 0.8177) or high-grade ICANS (20% vs. 14%; *p* = 0.5624) in patients who received early corticosteroids vs. patients who received tocilizumab only was observed. Multivariable analysis showed that early corticosteroid application had no significant impact on OR for ICANS (OR 0.75; *p* = 0.7562). These results support previous findings disproving increased risk of ICANS related to early use of tocilizumab [17,19,20]. However, these data also contradict any hypothesized prophylactic effect of corticosteroids on subsequent neurotoxicity [7,18,23].

Moreover, our study provides relevant data supporting the fact that the early use of corticosteroids does not impact the treatment efficacy of CAR T-cell therapy. No changes in outcome of CAR T-cell therapy due to prophylactic steroid applications were observed, congruent with previous data [7,17,18,23,24,25]. ORRs (80% vs. 77%; *p* = 0.7936) and CRRs (50% vs. 44%; *p* = 0.6628) were comparable between the Tcz and EcsTcz cohorts. Curve comparisons and median survival data were similar for PFS (*p* = 0.6345) and OS (*p* = 0.1215). In the multivariable analysis, early corticosteroid application showed no impact on HR for PFS (HR 0.56; *p* = 0.2737) or OS (HR 1.29; *p* = 0.6531). Relevantly, with an over-2-year follow-up, median OS was 36.5 months for the Tcz cohort, which is in line with previously published trial results [3,40].

This study was limited in that it was of a single-center retrospective cohort design. Thus, the safety and potential benefits of early corticosteroid use following CAR T-cell therapy should be further investigated in prospective randomized interventional studies. Moreover, recently proposed predictive scores for CRS and ICANS, integrating laboratory and clinical data, might be used to find optimal prophylactic and/or early corticosteroid regimens in order to prevent high-grade CRS and ICANS [25,41,42]. Finally, recent data on the underlying mechanisms of CAR T-mediated neurotoxicity have suggested that CAR T-cells may allow other cytotoxic T-cells across the blood–brain barrier and that corticosteroids only insufficiently antagonize this T-cell infiltration [43]. Further translational research is needed to provide mechanistic insights and uncover novel therapeutic strategies for more effective prevention and management of ICANS.

## 5. Conclusions

This retrospective cohort study assessed how the early application of corticosteroids for low-grade CRS correlates with the incidence of high-grade CRS and ICANS following CAR T-cell therapy in hematologic malignancies. The results from our study suggest that the early use of corticosteroids, in combination with standard tocilizumab, may significantly reduce progression from low- to high-grade CRS, without worsening the incidence and severity of ICANS or compromising the outcome of CAR T-cell therapy.

## Figures and Tables

**Table 1:** Comparison of baseline clinical characteristics before CAR T-cell therapy in the Tcz vs. EcsTcz cohorts.

Parameter	Tcz	EcsTcz	*p*-Value
Total, *n*	40	43	
Female/male sex (ratio)	21/19 (1.11)	24/19 (1.26)	0.8273
Age at diagnosis, median (range)	63 (20–76)	59 (17–79)	0.3957
Age at CAR T-cell infusion, median (range)	68 (25–79)	64 (18–82)	0.3387
Diagnosis			
DLBCL, *n* (%)	39 (98)	39 (91)	0.3612
Transformed DLBCL, *n* (%)	17 (43)	12 (28)	0.1765
Transformed from FL, *n* (%)	13 (33)	6 (14)	0.0660
Transformed from B-CLL/SLL, *n* (%)	3 (8)	2 (5)	0.6685
Transformed from MZL, *n* (%)	1 (3)	2 (5)	>0.9999
Transformed from BL, *n* (%)	0 (0)	2 (5)	0.4946
B-ALL (%)	1 (2)	4 (9)	0.3612
Initial Stage (a)			
I, *n* (%)	1 (3)	1 (2)	>0.9999
II, *n* (%)	3 (8)	10 (23)	0.0697
III, *n* (%)	7 (18)	5 (12)	0.5395
IV, *n* (%)	17 (43)	15 (35)	0.5063
Unknown	11 (28)	8 (28)	
Treatment lines before CAR T-cell therapy			
2 lines, *n* (%)	21 (53)	28 (65)	0.2710
3 lines, *n* (%)	12 (30)	8 (19)	0.3054
More than 3 lines, *n* (%)	4 (10)	3 (7)	0.7063
Unknown	3 (7)	4 (9)	
SCT, *n* (%)	27 (68)	16 (37)	0.0083
Autologous SCT (ASCT), *n* (%)	24 (60)	15 (35)	0.0285
Allogenic SCT, *n* (%)	3 (8)	1 (2)	0.3481
Bridging chemotherapy, *n* (%)	11 (28)	20 (47)	0.1114
Bridging radiotherapy, *n* (%)	5 (13)	11 (26)	0.1683
Remission status before CAR T-cell infusion			
CR, *n* (%)	1 (3)	4 (9)	0.3612
PR, *n* (%)	9 (23)	12 (28)	0.6207
SD, *n* (%)	9 (23)	11 (26)	0.8013
PD, *n* (%)	20 (50)	16 (37)	0.2734
Unknown	1 (1)	0 (0)	
CNS involvement, *n* (%)	4 (10)	7 (16)	0.5226
LDH elevation before CAR T-cell infusion, *n* (%)	9 (23)	5 (12)	0.2448
Months from diagnosis to CAR T-cell infusion, median (range)	61 (6–330)	60 (6–311)	0.9460

Abbreviations: ASCT: autologous stem cell transplantation; B-ALL: B-lineage acute lymphoblastic leukemia; B-CLL: B-cell chronic lymphatic leukemia; BL: Burkitt lymphoma; CNS: central nervous system; CR: complete response; DLBCL: diffuse large B-cell lymphoma; EcsTcz: patients who received early corticosteroid applications and tocilizumab; FL: follicular lymphoma; LDH: lactate dehydrogenase; MZL: marginal zone lymphoma; PD: progressive disease; PR: partial response; SCT, stem-cell transplantation; SD: stable disease; SLL: small lymphocytic lymphoma; Tcz: patients who received tocilizumab only (without early corticosteroids). (a) For DLBCL according to the Ann Arbor staging system, B-ALL excluded.

**Table 2:** Comparison of therapy details and laboratory values during hospitalization period for CAR T-cell therapy in cohorts Tcz vs. EcsTcz.

Parameter	Tcz	EcsTcz	*p*-Value
Total, *n*	40	43	
CAR T-cell product			
Tisagenlecleucel (Kymriah^®^), *n* (%)	29 (73)	29 (67)	0.6406
Axicabtagene-Ciloleucel (Yescarta^®^), *n* (%)	11 (27)	14 (33)	
Early corticosteroids, *n* (%)	0 (0)	31 (72)	<0.0001
Cumulative dose in mg, median (range)	-	40 (10–40)	
Tocilizumab (Actemra^®^), *n* (%)	20 (50)	32 (74)	0.0253
Days from CAR T-cell infusion to application of first tocilizumab dose, median (range)	3 (0–12)	2 (0–9)	0.5114
Number of applications, median (range)	4 (3–6)	4 (1–8)	0.1017
Cumulative dose in mg, median (range)	3200 (1800–4800)	2400 (400–4000)	0.0054
Use of filgrastim (G-CSF), *n* (%)	25 (63)	17 (100)	<0.0001
Subsequent corticosteroids (a), *n* (%)	12 (30)	17 (40)	0.4899
Dose escalation of subsequent corticosteroids (b), *n* (%)	3 (8)	6 (14)	0.4835
Duration of subsequent corticosteroids, days, median (range)	27 (1–208)	29 (12–98)	0.8359
Cumulative dose of subsequent corticosteroids in mg, median (range)	428 (30–1640)	492 (150–1116)	0.3956
Application of siltuximab (Sylvant^®^), *n* (%)	7 (18)	5 (12)	0.5395
Peak CRP, mg/L, median (range)	57 (3–328)	27 (3–272)	0.1006
Days from CAR T-cell infusion to peak CRP, median (range)	3 (0–98)	3 (0–12)	0.1662
Peak IL-6, pg/mL, median (range)	179 (7–157,117)	677 (4–16,863)	0.8615
Days from CAR T-cell infusion to peak IL-6, median (range)	4 (0–98)	4 (1–32)	0.6612
Peak IL-1β, pg/mL, median (range)	0 (0–74.2)	0.5 (0–13.9)	0.4752
Days from CAR T-cell infusion to peak IL-1β, median (range)	9 (4–21)	5.5 (0–87)	0.9791
Peak ferritin, median (range)	1450 (99–12,398)	1209 (175–35,199)	0.5234
Days from CAR T-cell infusion to peak ferritin, median (range)	8 (0–44)	10 (0–45)	0.5249
Peak expansion of CAR T-cells, copies/μg DNA, median (range)	4636 (54–127,942)	3227 (37–49,166)	0.2645
Days to peak expansion of CAR-T cells, median (range)	10 (1–37)	9 (5–22)	0.4157

Abbreviations: CRP: C-reactive protein; G-CSF: granulocyte-colony-stimulating factor; IL-6: interleukin-6; IL-1β: interleukin-1β. (a) Tocilizumab subsequent to corticosteroids excluding the investigated early corticosteroids. (b) Maximal dose of corticosteroid therapy higher than starting dose.

**Table 3:** Comparison of adverse events from CAR T-cell therapy in cohorts Tcz vs. EcsTcz.

Parameter	Tcz	EcsTcz	*p*-Value
Total, *n*	40	43	
CRS			
All grades (1–4), *n* (%)	28 (70)	39 (91)	0.0249
High-grade (3–4), *n* (%)	4 (10)	0 (0)	0.0497
Grade 1, *n* (%)	13 (33)	29 (67)	0.0021
Grade 2, *n* (%)	11 (28)	10 (23)	0.8013
Grade 3, *n* (%)	3 (8)	0 (0)	0.1075
Grade 4, *n* (%)	1 (3)	0 (0)	0.4819
Days from CAR T-cell infusion until CRS, median (range)	3.5 (0–12)	2 (0–13)	0.2547
Rebound CRS, *n* (%)	0 (0)	3 (7)	0.2418
ICANS			
All grades (1–4), *n* (%)	12 (30)	14 (33)	0.8177
High-grade (3–4), *n* (%)	8 (20)	6 (14)	0.5624
Grade 1, *n* (%)	3 (8)	4 (9)	>0.9999
Grade 2, *n* (%)	1 (3)	5 (12)	0.2033
Grade 3, *n* (%)	6 (15)	5 (12)	0.7515
Grade 4, *n* (%)	2 (5)	1 (2)	>0.9999
Days from CAR T-cell infusion until ICANS, median (range)	5.5 (1–15)	6 (2–11)	0.9474
Rebound ICANS, *n* (%)	2 (5)	2 (5)	>0.9999
CARTOX-10 score			
10, *n* (%)	27 (73)	32 (74)	>0.9999
7–9, *n* (%)	1 (3)	5 (12)	0.2089
3–6, *n* (%)	0 (0)	2 (5)	0.4965
1–2, *n* (%)	2 (5)	1 (2)	0.5933
0, *n* (%)	7 (19)	3 (7)	0.1743
Days until lowest score, median (range)	7 (1–29)	9 (3–37)	0.7500
Days until recovery, median (range)	2 (0–8)	1 (0–16)	0.9378
Additional diagnostics due to ICANS, *n* (%)	12 (30)	11 (26)	0.8067
MRT, *n* (%)	1 (3)	7 (16)	0.0586
CT, *n* (%)	3 (8)	2 (5)	0.6685
MRT and CT, *n* (%)	8 (20)	4 (9)	0.2173
Abnormalities in MRT and CT, *n* (%)	2 (5)	2 (5)	>0.9999
Abnormalities in EEG, *n* (%)	2 (5)	7 (16)	0.1579
ICU transfer, *n* (%)	8 (20)	5 (12)	0.3709
Days of hospitalization, median (range)	22 (14–52)	21 (16–46)	0.7611

Abbreviations: CARTOX: CAR T-cell-therapy-associated toxicity; CRS: cytokine release syndrome; CT: computer tomography; EEG: electroencephalogram; ICANS: immune-effector-cell-associated neurotoxicity syndrome; ICU: intensive care unit; MRT: magnet resonance tomography.

**Table 4:** Correlation of multiple predictors with the incidence of CRS and ICANS during CAR T-cell therapy.

	CRS		ICANS	
Predictors	Multivariable OR * (95% CI)	*p*-Value	Multivariable OR * (95% CI)	*p*-Value
Male sex	1.92 (0.53–7.72)	0.3285	0.65 (0.14–2.70)	0.5539
Age > median (61 years)	1.74 (0.31–10.52)	0.5428	10.16 (1.86–74.91)	0.0123
Transformed DLBCL	0.71 (0.17–3.10)	0.6237	0.39 (0.06–2.14)	0.2920
Initial stage at diagnosis	0.88 (0.35–1.94)	0.8244	1.57 (0.72–3.65)	0.2621
Lines of therapy before CAR T-cell therapy	0.82 (0.41–1.55)	0.5857	1.11 (0.40–2.74)	0.8315
ASCT	1.06 (0.21–5.56)	0.9593	1.84 (0.34–11.51)	0.4872
Bridging chemotherapy	1.61 (0.41–7.19)	0.5021	0.74 (0.17–2.89)	0.6743
Bridging radiotherapy	4.88 (0.60–118.75)	0.2092	0.52 (0.05–4.35)	0.5578
Remission status before CAR T-cell infusion	1.20 (0.57–2.57)	0.6419	1.47 (0.63–3.88)	0.3940
CNS involvement	1.25 (0.17–13.95)	0.8409	1.70 (0.26–11.44)	0.5722
LDH elevation before CAR T-cell infusion	0.55 (0.09–3.00)	0.4851	1.19 (0.18–7.63)	0.8503
Axicabtagene-ciloleucel vs. tisagenlecleucel	0.41 (0.08–2.01)	0.2716	1.00 (0.20–4.79)	0.9997
G-CSF application	10.29 (1.90–73.67)	0.0107	2.27 (0.27–33.14)	0.4689
Early corticosteroid application	-	-	0.75 (0.12–4.46)	0.7562
High-grade CRS	-	-	0.70 (0.04–13.25)	0.8113
IL-6 > median (41 mg/l)	-	-	5.11 (0.99–33.14)	0.0620
CRP > median (44 pg/l)	-	-	3.32 (0.81–15.83)	0.1071
Ferritin > median (1257 pg/l)	-	-	2.80 (0.65–13.32)	0.1736
IL1-β > median (0.45 μg/mL)	-	-	1.54 (0.27–9.19)	0.6220

Abbreviations: CI: confidence interval; OR: odds ratio. * Adjusted for all other variables.

**Table 5:** Comparison of outcomes of CAR T-cell therapy in the Tcz vs. EcsTcz cohorts.

Parameter	Tcz	EcsTcz	*p*-Value
Total, *n*	40	43	
Best response			
CR, *n* (%)	20 (50)	19 (44)	0.6628
PR, *n* (%)	12 (30)	14 (33)	0.8177
SD, *n* (%)	2 (5)	4 (9)	0.6770
PD, *n* (%)	2 (5)	4 (9)	0.6770
Remission status at last follow-up			
CR, *n* (%)	20 (50)	18 (42)	0.5126
PR, *n* (%)	7 (18)	8 (19)	>0.9999
SD, *n* (%)	0 (0)	2 (5)	0.4946
PD, *n* (%)	9 (23)	12 (28)	0.6207
Overall response rate	32 (80)	33 (77)	0.7936
Complete response rate	20 (50)	19 (44)	0.6628
PFS			
Median survival, months, curve comparison	17.6	11.4	0.6345
Median follow-up, months	26.6	6.25	
PFS at 12 months			
Median survival, months, curve comparison	n/a	11.41	0.8221
Median follow-up, months	12	6.25	
OS			
Median survival, months, curve comparison	36.49	10.98	0.1215
Median follow-up, months	25.22	9.44	
OS at 12 months			
Median survival, months, curve comparison	n/a	10.98	0.3201
Median follow-up, months	12	9.44	

Abbreviations: CR: complete response; PD: progressive disease; PFS: progression-free survival; PR: partial response; OS: overall survival; SD: stable disease.

**Table 6:** Correlation of multiple predictors with PFS and OS after CAR T-cell therapy.

	PFS		OS	
Predictors	Multivariable HR * (95% CI)	*p*-Value	Multivariable * HR (95% CI)	*p*-Value
Male sex	3.35 (1.29–8.75)	0.0134	1.39 (0.61–3.18)	0.4290
Age > median (61 years)	0.32 (0.09–1.17)	0.0854	2.50 (0.81–7.68)	0.1102
Transformed DLBCL	1.47 (0.52–4.18)	0.4724	0.37 (0.11–1.20)	0.0972
Initial disease stage at diagnosis	0.62 (0.38–0.99)	0.0475	1.11 (0.71–1.75)	0.6385
Treatment lines before CAR T-cell therapy	1.14 (0.74–1.76)	0.5450	1.17 (0.41–2.96)	0.4406
ASCT	0.41 (0.14–1.20)	0.1036	1.05 (0.42–2.64)	0.9111
Bridging chemotherapy	2.44 (0.98–6.08)	0.0554	2.06 (0.99–4.29)	0.0526
Bridging radiotherapy	0.50 (0.16–1.62)	0.2499	0.76 (0.24–2.43)	0.6476
Remission status before CAR T-cell infusion	1.60 (0.95–2.69)	0.0780	1.24 (0.72–2.13)	0.4403
CNS involvement	0.41 (0.06–2.65)	0.3465	0.87 (0.28–2.69)	0.8147
LDH elevation before CAR T-cell infusion	0.30 (0.08–1.13)	0.0745	0.86 (0.33–2.20)	0.7490
Axicabtagene-Ciloleucel vs. Tisagenlecleucel	0.46 (0.19–1.10)	0.0792	0.31 (0.12–0.77)	0.0122
G-CSF application	5.09 (1.12–23.05)	0.0348	2.04 (0.44–9.44)	0.3621
Early corticosteroid application	0.56 (0.20–1.58)	0.2737	1.29 (0.43–3.85)	0.6531
Subsequent corticosteroid application	4.67 (0.83–26.13)	0.0795	4.03 (1.08–14.99)	0.0374
CRS	0.35 (0.08–1.42)	0.1402	0.73 (0.16–3.29)	0.6786
ICANS	0.40 (0.09–1.88)	0.2486	0.60 (0.18–2.01)	0.4122
Duration of hospitalization	1.06 (0.98–1.14)	0.1562	1.02 (0.96–1.09)	0.5199
IL-6 > median (41 mg/l)	1.79 (0.37–8.71)	0.4700	1.05 (0.35–3.13)	0.9251
CRP > median (44 pg/l)	1.73 (0.72–4.15)	0.2199	1.79 (0.77–4.17)	0.1738
Ferritin > median (1257 pg/l)	2.91 (1.03–8.17)	0.0430	1.70 (0.71–4.09)	0.2326
IL1-β > median (0.45 μg/mL)	0.67 (0.21–2.16)	0.4978	1.03 (0.37–2.88)	0.9556

Abbreviations: HR, hazard ratio. * Adjusted for all other variables.

## Data Availability

No data supporting the reported results are deposited elsewhere.
